# Targeting Mechanotransduction in Osteosarcoma: A Comparative Oncology Perspective

**DOI:** 10.3390/ijms21207595

**Published:** 2020-10-14

**Authors:** Anita K. Luu, Alicia M. Viloria-Petit

**Affiliations:** Department of Biomedical Sciences, Ontario Veterinary College, University of Guelph, Guelph, ON N1G 2W1, Canada; aluu@uoguelph.ca

**Keywords:** osteosarcoma, mechanotransduction, comparative oncology, ezrin, Hippo signalling, TAZ, YAP, myocardin-related transcription factor-A/-B, molecular targeted therapy

## Abstract

Mechanotransduction is the process in which cells can convert extracellular mechanical stimuli into biochemical changes within a cell. While this a normal process for physiological development and function in many organ systems, tumour cells can exploit this process to promote tumour progression. Here we summarise the current state of knowledge of mechanotransduction in osteosarcoma (OSA), the most common primary bone tumour, referencing both human and canine models and other similar mesenchymal malignancies (e.g., Ewing sarcoma). Specifically, we discuss the mechanical properties of OSA cells, the pathways that these cells utilise to respond to external mechanical cues, and mechanotransduction-targeting strategies tested in OSA so far. We point out gaps in the literature and propose avenues to address them. Understanding how the physical microenvironment influences cell signalling and behaviour will lead to the improved design of strategies to target the mechanical vulnerabilities of OSA cells.

## 1. Introduction

The bone is a dynamic tissue that provides structural support to the human body throughout one’s life; as such, it must be able to undergo adaptive processes to maintain structural integrity during various mechanical stimuli, such as walking and running [1]. The ability of bone to respond to mechanical loading has been known for centuries. In 1892, the German surgeon, Dr. Julius Wolff, published his work commonly referred to as ‘Wolff’s Law’, which combined the anatomical drawings of Meyer, and principles by Bourgery or Bell (unknown at time) and Roux [2,3]. Although Roux’s principle postulated that local bone cells regulate the mechanical response, the majority of Wolff’s Law attempted the use of mathematical rules to explain how the trabecular architecture is positioned to withstand mechanical forces. It was highly criticised because of insufficient evidence and its inadequacy in addressing the biological basis of bone organisation [3,4]. The link between biology and mechanical force was not proposed until almost a century later, when Harold Frost postulated the ‘mechanostat theory’ [5]. This theory stated that a mechanical threshold needs to be reached in order to activate a bone modelling or remodelling response [5,6]. To elicit this response, bone must generate a mechanical signal when loaded, which is detected by cells, leading to a secondary signal and thus either an increase or decrease in bone mass. Frost also hypothesised that different factors such as disease, hormones, and biochemical messengers could change the mechanical setpoint of bone. Although not known at the time, Frost’s theory attempted to explain the process we now refer to as mechanotransduction.

## 2. Mechanotransduction in Bone

Mechanotransduction is broadly defined as the ability of cells to convert mechanical stimuli from its surrounding environment into biochemical signals [7]. Despite being a general term, the process of mechanotransduction is a multi-step process that includes: (1) mechanocoupling, the reception of the mechanical signal by the sensor cell; (2) biochemical coupling, the translation of the mechanical signal into a biochemical signal (protein activation and/or gene expression), allowing for the (3) transfer of signal to the effector cell; leading to a (4) cell response [8,9]. In the context of the bone tissue, the most obvious and specialised candidate to receive and transmit mechanical cues is the osteocyte [6,10,11]. Osteocytes are the most abundant type of bone cell, making up approximately 90–95% of total cells within bone tissue [12]. Osteocytes are mature osteoblasts that are completely surrounded by a mineralised bone matrix. The cell body of an osteocyte resides within a lacuna, while several cytoplasmic processes, also referred to as dendrites, protrude outwards through small tunnel-like structures called canaliculi. The lacuna-canalicular network is a high-order system; on average, osteocytes have 89 projections per cell body which are ~47 micrometers in length and can branch approximately 12.7 times. This intricate neuron-like network allows communication between osteocytes, between osteocytes and other bone cells, such as osteoblasts and osteoclasts, as well as the exchange of materials within vascular pores [13,14]. 

Interestingly, the skeleton as a whole is believed to be subjected only to a minuscule amount of strain overall (approximately 0.04–0.3%) compared to the levels needed to elicit an effect on bone cells in vitro (1–10%) [10,15,16]. Thus, in order for osteocytes to respond to a mechanical stimulus, the mechanical signal must be amplified. Computational modelling predicts that osteocytes can amplify applied strains, equivalent to vigorous exercise, by approximately 350–400%. At higher magnitudes of strain, the surrounding pericellular matrix can increase the cell volume by 4–10%, while the extracellular matrix can amplify the strain by 50–420% [17]. This leads to a subsequent increase in interstitial fluid velocity and shear stress within canaliculi [18], which is predicted to increase with increasing mechanical loads [19]. Osteoblasts are more receptive to fluid shear stress than mechanical stress, suggesting that an increase in osteocyte fluid flow could be advantageous in osteoblast stimulation [20].

As the primary mechanosensor of the bone, the role of osteocytes appears to be two-fold: (1) increase the mechanical signal and (2) secrete signalling factors and other mediators to regulate the response of other bone cells (osteoblasts and osteoclasts). In response to mechanical stimuli, such as fluid flow, osteocytes have been shown to rapidly increase the release of prostaglandin E_2_ (PGE_2_) and cyclooxygenase 2 (COX2), the levels of Ca^2+^, and secondary messengers adenosine triphosphate (ATP) and cyclic adenosine monophosphate (cAMP), through direct and indirect mechanisms. Fluid flow has been shown to directly increase the levels of COX2 mRNA and the release of PGE_2_ [21]. This PGE_2_ acts in an autocrine manner by binding to EP_2_ receptors to increase both cAMP-dependent protein kinase A (PKA) signalling and phosphatidylinositol 3-kinase (PI3K)/Akt signalling [22,23]. As a result of pathway activation, glycogen synthase kinase 3 (GSK3) is inactivated, causing the accumulation of β-catenin in the nucleus, leading to the transcription of connexin 43 (CX43), an important protein in the formation of gap junctions. These gap junctions are important for maintaining the physical connection between and within bone cell types, allowing coordination of cell behaviour and promoting bone formation upon mechanical stimulus [24,25].

Aside from PGE_2_ release, another early response to mechanical signalling in osteocytes is the rapid increase in intracellular (cytoplasmic) Ca^2+^ levels. This increase in Ca^2+^ is mediated by various signalling molecules, pathways and cellular compartments and involves the activity of primary ciliary protein transient receptor potential vanilloid subfamily member 4 (TRPV4), the opening of T-type voltage-sensitive calcium channels, the controlled release and refilling of intracellular calcium stores in the endoplasmic reticulum (ER), P2 purinergic receptor (P2R)/phospholipase C (PLC)/inositol trisphosphate/ER pathway activation, sphingosine-1-phosphate signalling and the binding of matrix molecules to α_3_β_v_ integrins [26,27,28,29,30,31]. The influx of Ca^2+^ in osteocytes is important for the release of ATP, which in turn modulates actin dynamics and extracellular vesicle release [32,33]. In response to fluid flow, increases in intracellular Ca^2^ result in actin contractions though non-muscle myosin and myosin light chain kinase (MLCK), leading to an increase in extracellular vesicle (EV) release. These vesicles contain receptor activator of nuclear factor kappa-B ligand (RANKL), osteoprotegerin (OGN) and sclerostin, suggesting that its contents can regulate the bone formation process [33].

Not only are the dendritic process of osteocytes important for forming the junctions between cells, but they have shown to have roles in amplifying the mechanical signal. Thi and colleagues (2013) found that dendritic processes are very responsive to mechanical loading compared to the cell body and can rapidly increase the levels of intracellular Ca^2+^ [34]. This increase within the cell processes requires firm attachment to matrix through integrin α_v_β_3_ binding, as disruption of these adhesion sites prevented Ca^2+^ influxes. Mechanical loading on dendritic processes can lead to the opening of hemichannels on the cell body. This is dependent on glycocalyx that surrounds the dendritic processes. Abolishment of glycocalyx decreases the expression of α_5_ integrin and a subsequent decrease in hemichannel opening in osteocyte cell bodies upon mechanical stimulation [35]. This suggests that glycocalyx maintains the level of α_5_ integrin to respond and transmit mechanical cues along the osteocyte.

**Figure 1:**
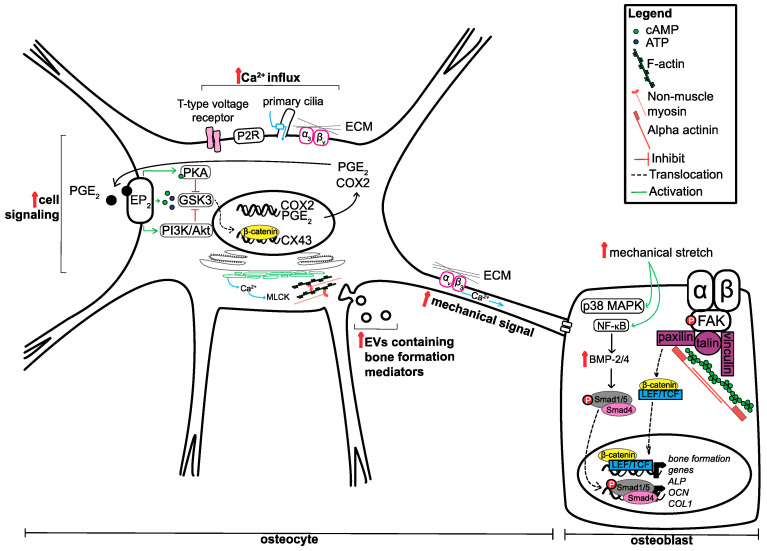
Summary of mechanisms utilised by osteocytes and osteoblasts to respond to mechanical cues. Although both osteocytes and osteoblasts utilise similar mechanisms to respond to mechanical cues, the primary outcome is not the same. Osteocytes respond to mechanical stimuli by increasing secondary messengers and the mechanical signals for neighbouring cells, including osteoblasts. Osteoblasts utilise these signals to increase the expression of genes involved in bone formation through the translocation of various transcription factors, mediated by signalling pathways and mechanically responsive proteins. Upward pointing red arrow indicates increase.

Although osteocytes are regarded as the mechanosensors of the bone, osteoblasts themselves have been widely demonstrated to be able to receive and respond to mechanical stimuli. One model proposed by Pavalko and colleagues (2003) suggests that osteoblasts activate gene transcription in response to mechanical loading through the formation of ‘mechanosomes’ [36]. Upon mechanical loading, changes within the cell membrane cause the organisation and recruitment of integrins, and focal adhesion complex proteins, such as focal adhesion kinase (FAK) and the Ras homologue (Rho) guanosine triphosphatase (GTPase), as well as binding of alpha-actinin to the cytoskeleton, and release of β-catenin from adherens junctions. The recruitment of these key signalling factors allows the formation of protein complexes that are able to translocate to the nucleus to activate gene transcription. In this model, it is predicted that cytoplasmic β-catenin associates with lymphoid enhancer-binding factor/T-cell factor (LEF/TCF), an architectural transcription factor, able to change shape of regulatory regions of target genes. This ‘bending of the genes’ can promote the transcription of bone formation-related genes, although the exact genes were not discussed in the model [36]. This model has been revisited in 2010 to add additional evidence in its support, however little is still known about how, and if, it can modulate the activity of other signalling pathways [37]. The elucidation of the relevance of this model in relation to other signalling pathways will be informative, as several pathways have been described to be activated in response to mechanical stimulation. For instance, osteoblast subjected to mechanical stretch activate both the p38 mitogen-activated protein kinase (p38 MAPK) and nuclear factor kappa B (NF-kappa B) signalling pathways. This leads to the upregulation of bone morphogenetic proteins, BMP-2 and BMP-4, activation of downstream small mothers against decapentaplegic (SMAD) signalling, and subsequent increase in alkaline phosphatase (*ALP*), osteocalcin (*OCN*) and collagen I (*COLI*) mRNA and protein levels [38,39]. Similarly, integrin alpha v beta 1 engagement has been shown to increase Src and Jun N-terminal kinase (JNK) signalling and beta-catenin nuclear localisation, respectively, as well as *ALP* and *OCN* mRNA expression [40,41]. The discussed mechanisms that both osteocytes and osteoblasts use to respond to mechanical signals are summarised in Figure 1.

Although mechanotransduction is a process that is often used by the skeletal system to maintain homeostasis, pathologies such as cancer, can exploit this process to aid in cancer progression [7]. 

## 3. Osteosarcoma

Osteosarcoma (OSA) is the most common primary cancer of the bone in both humans and canines. In humans, the disease most commonly affects adolescents, while middle-to-older aged canines are impacted [42,43,44]. Histologically, OSA is characterised by transformed bone progenitor cells that produce immature osteoid, while radiographically, it presents as a ‘sunburst’ appearance or triangular appearance (‘Codman’s Triangle’) due to the osteolytic or osteoblastic nature, and the bone lesion’s ability to elevate the periosteal surface, respectively [45,46]. The disease most commonly presents in the metaphyseal region of long bones of the limb such as the femur, tibia or humerus [47]. The most common symptom of OSA is pain and lameness of the impacted limb which can sometimes lead to pathological fracture. OSA diagnosis typically includes a combination of radiographic evidence, clinical presentation, and histopathology. Histopathological analysis of the bone specimen, obtained from open or closed sampling techniques, is performed to confirm diagnosis [48]. Human OSA is usually graded and staged using the Enneking/Musculoskeletal Tumour Society (MSTS) and American Joint Committee on Cancer (AJCC) systems [49]; while two methods have been proposed by Kirpensteijn [50] and Loukopoulos [51] for canine OSA. However, neither method proposed for canine OSA adequately predicts outcomes for dogs undergoing standard of care [52]. It is also important that patients undergo thoracic radiography to determine the extent of metastatic disease [53].

Treatment for both species includes the surgical removal of the tumour, either through limb amputation or limb-spare/limb-salvage surgery and adjuvant chemotherapy [54]. The most common chemotherapy agents used to treat OSA include doxorubicin, cisplatin and carboplatin (for both species), while ifosfamide (canine OSA) and lobaplatin and methotrexate (human OSA) might also be employed [55,56,57]. The addition of adjuvant chemotherapy has greatly increased the survival rate and the overall prognosis of both human and canine patients through delaying metastasis [57,58,59]. The most common location for OSA metastasis is the lung, and it is suspected that most patients have subclinical metastasis at the time of diagnosis [60]. With conventional therapy, localised OSA has a 5-year survival rate of approximately 60–70%, while this rate drops to ~20% when patients present with metastatic disease [61,62]. Given that the current treatment protocol for OSA management has remained unchanged in human and OSA patients in 30 years, and has been ineffective in treating metastatic OSA, it is imperative that we utilise comparative models to understand OSA metastasis biology and to accelerate the discovery of improved treatments for both human and canine patients.

While osteosarcoma is a rare disease in humans, and is often considered an ‘orphan disease’, it affects canines at a higher (up to 14-fold, depending on the breed population) incidence rate [63]. Research in both canine and human OSA shows that similar pathways are dysregulated, and comparison of the gene profiles of human and canine OSA samples show that both species share similar genetic abnormalities that cannot be distinguished [64]. The increased knowledge of both human and canine OSA biology has improved our understanding of mechanisms that aid in tumour growth and metastatic progression, suggesting alternative molecular targeted therapies. In general, targeted therapy approaches include the use of antibodies or small molecule inhibitors that specifically block the activity of a protein of interest by different mechanisms [65]. Most of the targeted therapies for both canine and human osteosarcoma to date have focused on signalling pathways that appear to be overactive in OSA tumours, such as human epidermal growth factor receptor 2 (HER2), insulin-like growth factor 1 (IGF1) and mammalian target of rapamycin (mTOR) signalling [66]. Another promising avenue is the targeting of molecules involved in mechanotransduction, the rationale and potential of which we discuss in the following sections.

## 4. Mechanotransduction in Cancer

The microenvironment that surrounds cancer cells, or tumour microenvironment, has been widely accepted to drive cancer progression. The microenvironment not only encompasses the classical signalling factors, but also includes the physical environment [67]. Research conducted in breast cancer has demonstrated that malignant breast lesions are stiffer than benign lesions and stiffness has predictive value for treatment response [68,69]. The increased stiffness observed in breast cancer lesions could be attributed to the changes in extracellular matrix (ECM) composition, including the increased deposition of collagen fibres, glycoproteins (fibronectin, tenascin, elastin), and/or the increased matrix crosslinking through the activity of lysyl oxidase (LOX) [70,71,72]. This increase in stiffness can be perceived by the cell at the cell-ECM interface through integrins and the formation of focal adhesions, which involve several proteins, such as talin and vinculin [73]. The assembly of these complexes allow for the activation of focal adhesion kinase (FAK) signalling, leading to cytoskeleton contractility and downstream activation of certain transcription factors and thus, gene expression. Studies in epithelial malignancies demonstrate that mechanotransduction can promote cancer cell proliferation, plasticity, chemoresistance, migratory, and metastatic properties and stemness [74,75,76,77,78].

## 5. Mechanotransduction in Osteosarcoma and Promising Targetable Pathways

The increasing sophistication of molecular biology tools has allowed researchers to understand mechanobiology in much greater detail in the last two decades. The use of hydrogels, water-based crosslinked polymers that can be easily manipulated to varying stiffnesses (Young’s module), have permitted the assessment of how this physical parameter impacts mechanotransduction in relation to various ECM components, while 3D culture models have allowed for better mimicking of the architecture of the tissue of interest. For example, a 3D collagen scaffold model created by Liverani and colleagues (2019), allowed for the culture of breast cancer cells in an environment that mimics the hierarchically organised structure of extracellular collagen in actual tissue. Furthermore, this model permits the study of hypoxia effects on cell fate and metabolism, as tissue-like cellular structures are able to establish realistic oxygen gradients, which contributes to their overall phenotype [79]. Another invaluable tool in the study of mechanobiology is atomic force microscopy, which permits the mechanical profiling of matrices and individual cells on a nanometre scale. Several other tools and techniques are currently used in the study of mechanobiology as well, but are out of the scope of this review and has been well discussed in a review by Mohammed and colleagues [80].

### 5.1. Mechanical Properties of OSA Cells

Mechanical characterisation of mesenchymal stem cells, normal osteoblasts and OSA cells demonstrated that there are different mechanical profiles between and within cell types. The human OSA cell line MG63, for instance, appeared to be softer because of less organised cytoskeleton and had a rougher surface than its normal counterparts [81]. These differences do not only exist between normal and malignant bone cells, but also can extend to OSA cells at different stages of malignancies. A study comparing two paired low metastatic and high metastatic variants of OSA cells demonstrated that low metastatic cells have a greater focal adhesion count and density in adhesion conditions, and unsurprisingly, generate greater traction forces [82]. Interestingly, this did not necessarily translate to an increased overall cell stiffness, or increased mechanosignalling, as determined by the activity of Rho-associated, coiled-coil containing protein kinase (ROCK). In free-flowing conditions, low metastatic cells have slightly increased stiffness and overall cell volume as compared to high metastatic cells, however there were no differences in the levels of nuclear matrix proteins, which has been shown to mediate changes in overall cell stiffness [82]. Although some findings were not consistent between the two paired cell lines, they are somewhat in line with what has previously been described for epithelial cancer cells, such as those of the breast, and could be explained by phenotype and behaviour of malignant cells. In order to metastasise, cancers that spread through the hematogenous route, such as OSA, must proceed through a variety of different steps, starting with invading the local stroma and intravasating into blood vessels [83]. A smaller cell volume and less organised cytoskeleton, as observed in the highly metastatic variant, may make cancer cells more flexible and able to accommodate small openings between endothelial cells of blood vessels [84,85]. Findings of a recent study by Rianna and colleagues (2020) support this hypothesis. When human OSA cells were exposed to Y-shaped channels that became progressively more confined, the Young’s moduli of the cell decreased from 5.6 kPa to 2.1 kPa [86]. Softer OSA cells can also be attributed to their differentiation state. An assessment of four cell lines that mimicked different stages within the bone differentiation program, i.e., undifferentiated mesenchymal stem cells, differentiated mesenchymal stem cells, osteoblasts and osteocytes, showed differences in cell shape and traction forces [87]. Interestingly, less differentiated cells had a smaller area, were more circular and had smaller levels of traction. Although this study was conducted in normal bone cells, the results lead one to predict that metastatic OSA cells behave like less differentiated normal bone cells. This also agrees with the proposed cell of origin for OSA, undifferentiated bone progenitor cells and the role that cancer stem cells play in tumour metastasis; perhaps the physical and mechanical features of metastatic cells reflects their reversion to a more stem cell-like, less differentiated phenotype [88,89]. The aforementioned studies (summarised in Table 1) provide a good initial characterisation of the mechanics of individual cancer cells, however additional studies with more cell lines or primary cells will be needed to understand this relationship further. The data on cell mechanics and cancer can be conflicting and depend on the area of measurement within the cell and its location in the cell cycle, adding another level of complexity in identifying a ‘mechanical signature’ to differentiate malignant from benign bone cells [90]. The overall stiffness of cancer cells may also be insufficient to explain their behaviour in different microenvironments.

### 5.2. Matrix Environments: Response to Environmental Stiffness

Studies employing techniques that mimic the bone environment or different matrix stiffnesses allow for a better understanding of the pathways involved in transducing mechanical cues in OSA and the role of mechanotransduction in OSA.

Perhaps not surprising, OSA cells have been shown to respond best to mechanical cues when the substrate rigidity is closest to that of its origin tissue. OSA cells isolated directly from patient tissue were found to have a greater cell area, traction forces and increased survival when seeded on stiffer substrates (55 kPa), which approximates the reported Young’s modulus of collagenous bone, versus soft substrates (1 or 7 kPa) [91]. Not only is the ‘bone-like’ stiffness important for OSA cell survival, but it has also been shown to be the optimal stiffness for the formation of cancer stem cells. Jabbari and colleagues encapsulated OSA cells in polyethylene glycol diacrylate (PEGDA) gels and found that they were most successful at forming CD44^+^ and CD133^+^ tumourspheres when the gel was 50 kPa in stiffness [92].

OSA cells utilise a variety of mechanisms to respond to mechanical stress, such as enhancing expression and/or activity of molecules that mediate mechanical signalling. As mentioned previously, integrins are responsible for receiving external mechanical stimuli. MG63 OSA cells exposed to cyclic mechanical stimulation upregulate their integrin beta 1, pFAK and pERK protein levels. The increases in pFAK and pERK levels were abrogated with an integrin beta 1 blocking antibody, demonstrating that this integrin is a key mediator of OSA mechanotransduction [93]. This capability of OSA cells to amplify mechanotransduction via increased integrin expression under conditions of mechanical stress might be important for varying responses to mechanical forces as compared to osteoblasts. When human OSA or osteoblasts were encapsulated in hydrogels with tunable stiffness and ECM adhesion ligand density, normal osteoblasts were more responsive to ECM adhesion ligand density changes to promote osteogenesis, while OSA cells were more responsive to stiffness changes. Increasing the stiffness of the hydrogel while keeping the ligand density constant led to an increase in proteins related to focal adhesion signalling, namely, integrin beta 1, talin-1, FAK, paxillin and vinculin and the increase in mRNA levels of downstream pro-tumourigenic factors: hypoxia inducible factor 1 alpha (HIF1 alpha), vascular endothelial growth factor (VEGF) and matrix metalloproteinase 2 and 9 (MMP2 and 9). When these hydrogel scaffolds were injected in vivo, the tumour volume was greatest in mice inoculated with hydrogel scaffolds with the greatest stiffness, providing evidence that stiffness impacts tumour growth in vivo [94].

Aside from transcription-dependent increases in the protein levels of several focal adhesion components, a study by Zheng and colleagues (2014) suggests that components of the ECM environment could be modified with mechanical stimuli as well as by similar or alternative mechanisms [95]. Tenascin-c is a glycoprotein located in the ECM environment that contains four different domains, one of which is the fibronectin III domain. Alternative splicing within the fibronectin repeat domain of tenascin-C permits the interaction with various growth factor receptors and proteins [96]. The fibronectin III A1 (FNIII A1) variant of tenascin-C, TN-C FNIII A1, is highly expressed in OSA tissue samples and can enhance cell migration in vitro when overexpressed. The mechanical stimulation of MG63 cells in a 3D collagen culture resulted in an increase in TN-C FNIII A1 mRNA which was blunted with mTOR inhibitors and knockdown of downstream mTOR signalling mediators eukaryotic translation initiation factor 4E (eIF4E)-binding protein 1 (4E-BP1) or p70 ribosomal protein S6 kinase 1 (S6K1) [95]. This study highlights how mechanically stimulated OSA cells can contribute to changes within the ECM environment, however there is little-to-no knowledge of how the ECM composition, or its mechanical properties, changes within the primary or secondary tumour microenvironment and the implication of such change for OSA progression.

The ECM contains structural proteins, (collagen type I, elastin), glycoproteins (vitronectin, fibronectin, laminin) and proteoglycans, with collagen being most abundant [97]. RNA analyses that compared paired non-tumour and tumour samples from human OSA patients, as well as paired primary and metastatic human and mouse OSA cell lines, indicate that ECM components are upregulated in tumour and metastatic variants [98,99,100]. Extensive areas of collagen deposition in metastatic lesions within the lung were also observed in a mouse model of OSA [101]. While this is expected from the ability of OSA cells to produce osteoid, it also indicates that metastatic OSA cells are readily able to modify their microenvironment, through increasing the levels of secreted collagen. Enhanced production of ECM modifying enzymes is another potential mechanism to alter ECM properties. Collagen synthesis is a multistep process that requires post-translational modifications through enzymatic activity at various residues: the prolyl 4-hydroxylases (P4Hs) are responsible for the formation of 4-hydroxyproline from proline to allow for proper folding of procollagen; the procollagen-lysine, 2-oxoglutarate 5-dioxygenase (PLODs), allows the hydroxylation of lysine residues to permit the intermolecular crosslinks between collagen molecules; and lysyl oxidase (LOX) converts lysine to aldehyde in collagens after secretion to allow crosslinking between collagens and between collagen and elastin (as reviewed in [102]). Interestingly, the expression of these enzymes is regulated by hypoxia, a common feature of the tumour microenvironment, in both cancer cells and normal fibroblasts.

Proteomic analysis comparing primary and metastatic canine OSA cells under normoxic or hypoxic conditions found that P4HA1, PLOD1 and 2 and LOX were all increased by hypoxia, with a more dramatic effect seen in the metastatic cell line [103]. Contrasting these observations, LOX was reported to have tumour suppressive effects in human OSA cell lines, although this capacity was not tested in vivo [104]. The results of this study in OSA are in disagreement to the well documented role of LOX in ECM remodelling in epithelial malignancies, leading to increased integrin signalling, and metastatic colonisation [105,106]. Similar involvement of LOX in tumour dissemination was reported for undifferentiated pleomorphic sarcoma (UPS), where HIF1 alpha promoted the expression of PLOD2, and PLOD2 inhibition led to a decrease in collagen deposition, organisation and maturation, and a decrease in lung metastases [107]. Further research is necessary to determine whether the same applies to OSA, and how these changes in ECM modulating enzymes can impact the mechanical microenvironment and mechanosignalling in this and other sarcomas.

**Figure 2:**
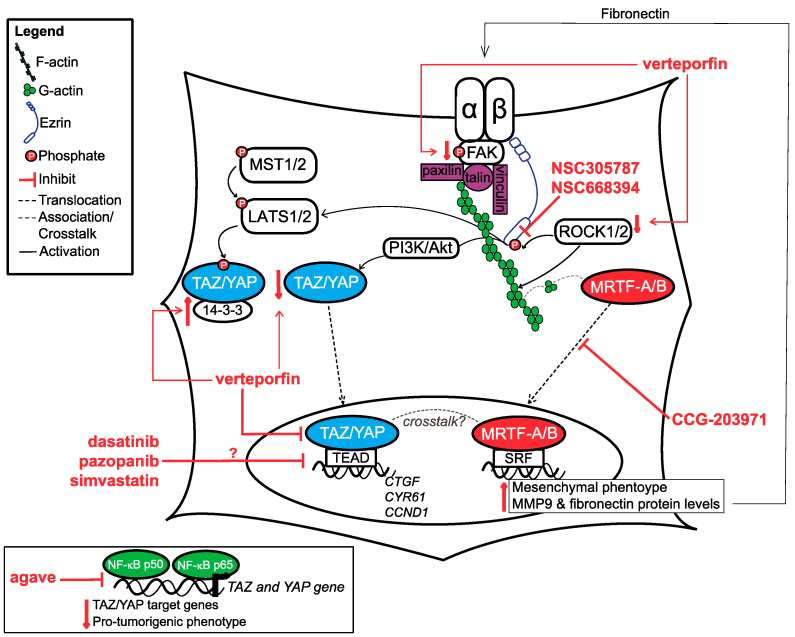
Summary of pathways that OSA cells used to respond to mechanical cues and therapeutic targets discussed in this manuscript. TAZ/YAP, MRTF-A/B and ezrin have all been demonstrated to be important for mechanical signalling in OSA. Upward pointing red arrow indicates increase, downward pointing red arrow indicates decrease.

In summary, OSA cells can respond to mechanical cues from their microenvironment by increasing the expression of ECM proteins and focal adhesion complex proteins (see Table 2 for a summary of all literature discussed). OSA cells can also modulate the composition and mechanical properties of the ECM, which in turn also alters the way they respond to mechanical stimulus. Altogether, these cellular adaptions lead to increased mechanical and thus, a more robust biochemical response. Key downstream players in this biochemical response are transcription factors, among which the Hippo signalling mediators TAZ (transcriptional co-activator with a PDZ-binding motif) and YAP (yes-associated protein) and MRTF-A/-B (myocardin-related transcription factor-A/-B) are worth mentioning. In particular, TAZ and YAP, have a consistently documented role in OSA progression. Another protein deserving our attention in regard to OSA is Ezrin, an adaptor and signalling molecule, and possibly a key hub in modulating transcriptional responses. The following section will discuss how all of the above-mentioned factors contribute to mechanotransduction-driven tumourigenic behaviour in OSA, and the various molecular targeting strategies explored until now to halter their activity (see Figure 2). Based on critical discussion of these strategies we hypothesise on their translational potential and propose paths to test this potential.

## 6. Hippo Pathway Mediators—TAZ/YAP

The Hippo pathway plays key roles in almost all organ systems—from embryonic development to adult tissue homeostasis and regeneration, as well as the development and advance of pathologies such as cancer [108,109]. Canonical Hippo signalling includes upstream kinases MST1/2 and LATS1/2, which ultimately mediate the localisation and function of downstream transcription factors TAZ and YAP (herein referred to as TAZ/YAP). The pathway itself is tumour suppressive as when this pathway is active, in which case LATS1/2 phosphorylate TAZ/YAP thus facilitating their retention in the cytoplasm, where they bind to 14-3-3 proteins and are subsequently degraded. Conversely, when this pathway is inactive, TAZ/YAP are able to translocate to the nucleus, where they bind to TEAD transcription factors to active gene transcription. In OSA, similar to other cancers, the TAZ/YAP-TEAD interaction has been shown to be essential for cell proliferation, invasion and survival [110]. Both TAZ and YAP are frequently upregulated in sarcoma malignancies [111]. In human OSA cell lines specifically, TAZ was shown to promote metastasis through miR224 [112], while YAP was demonstrated to mediate chemoresistance [113], and promote a cancer stem cell phenotype [114]. In addition, the nuclear expression of TAZ/YAP in human OSA tissue was shown to associate with a reduced progression-free survival [115]. The role of TAZ/YAP is similar in canine OSA, as depletion of TAZ or YAP in canine OSA cell lines significantly decreases cell migration and viability, and depletion of YAP enhances sensitivity to doxorubicin [116].

### 6.1. TAZ and YAP in OSA Mechanotransduction

Aside from upstream kinases MST1/2 and LATS1/2, several other mediators and environmental factors have been demonstrated to modulate TAZ/YAP expression, localisation and activity (see recent review by Pocaterra and colleagues [117]). TAZ/YAP have been highly regarded as mechanotransducers and can effectively respond to changes within the extracellular environment. TAZ/YAP activation process is driven by changes in cell morphology [118,119], actin processing factors [120], stress fibre formation, focal adhesion assembly [121] and the direct opening of nuclear pores upon stretch [122]. In a stiff environment, TAZ and YAP have demonstrated to be predominately nuclear and regulate the differentiation of mesenchymal stem cells into the osteoblast lineage [118,123,124].

Similar to what has been previously reported in the literature for osteoblasts differentiation [125], YAP nuclear localisation is highly influenced by cytoskeletal dynamics and associated mechanical signalling factors in OSA. Inhibition of actin polymerisation by cytochalasin D led to a decrease in nuclear YAP levels in MG63 human OSA cells [81]. Similarly, ROCK inhibition through siRNA or ROCK2 inhibitor SR3677, leads to a decrease in nuclear and overall YAP levels, and downstream target genes, cysteine-rich angiogenic inducer 61 (*CYR61),* connective tissue growth factor *(CTGF),* and cyclin D1 (*CCND1*). These findings are not solely limited to in vitro studies, as mice injected with ROCK-depleted OSA cells had a significantly decreased tumour volume and were unable to metastasise compared to mice injected with control OSA cells. Analysis of the tumour tissue isolated from mice injected with ROCK-depleted tumour cells demonstrated a decrease in total YAP immunolabeling, suggesting that modulation of YAP by ROCK might impact tumour growth and metastasis in OSA [126]. Not only is YAP important for tumour establishment and metastasis, its localisation is also dynamic throughout the tumour cell’s mechanical journey [127]. As mentioned previously, the overall stiffness of U2OS cells decrease as they move from less confined channels (100 micrometer in width) to more confined channels (5 micrometer in width). As U2OS cells move from these different environments, there are obvious changes in cell morphology and YAP localisation; predominately nuclear (less confined environment, where cells stiffen) to predominately cytoplasmic (more confined environment, where cells soften). Interestingly, it appears it is not just the change in cell morphology that influences YAP localisation, but rather the topographical (3D) confinements. When experiments were repeated using a 1D structure, U2OS cell morphology changes were observed while YAP remained nuclear throughout all conditions [86]. These results suggest that spatial changes with a 3D architecture are required to mediate changes in YAP localisation.

A similar rationale can be used to understand the results for a study by Molina and colleagues (2019), where MG63 cells were encapsulated within 3D meshes with varying concentrations of gelatin and poly (epsilon-caprolactone) (PCL), which mimics the porous structure of trabecular bone. Upon decreases in tensile moduli, YAP protein levels decrease, while TAZ levels stay consistent. Interestingly, the nuclear localisation of YAP and TAZ moderately increased as the tensile modulus decreases [128]. These findings are in contrast to what has been reported for TAZ/YAP but could be explained by the ECM structure in this model and the actin filament structure of the cells. The fibre meshwork and porosity of the matrix make it an advantageous model for understanding cell-to-cell interaction and exchange of nutrients or signalling factors but may be disadvantageous when trying to understand the physical forces surrounding a cell as the fibres are quite sparse [129]. Furthermore, despite the changes in the concentration of gelatin and PCL, the actin filaments of MG63 cells had a consistent morphology in all conditions. As actin filaments organisation is key to TAZ/YAP localisation, it makes sense that nuclear levels of TAZ/YAP were modestly impacted with a decreasing tensile modulus. These results may need to be examined further in comparison with other models to determine reproducibility and the relevance of each model to specific aspects of cancer biology.

### 6.2. Inhibitors of TAZ/YAP

As TAZ/YAP have well documented roles in human cancer progression, it is not surprising that they have generated significant therapeutic interest. Several different strategies have been employed to disrupt TAZ/YAP signalling, either through targeting their upstream mediators, directly targeting TAZ/YAP and/or TAZ/YAP outputs (see references [130,131,132]). For the purpose of this review, we only focused on inhibitors that have been tested in OSA, as discussed below.

#### 6.2.1. Verteporfin

Verteporfin, a small molecule benzoproyphyrin, is a light-activated drug currently used for the treatment of age-related macular degeneration. In the context of Hippo signalling, it is the only compound to date that has been shown to be able to bind to YAP. This prevents YAP binding to TEAD, and has been demonstrated to upregulate 14-3-3 binding proteins, thus causing YAP sequestration into the cytoplasm [133,134]. Studies completed in human OSA cells showed that verteporfin treatment led to a decrease in YAP levels, downstream YAP target genes and colony-forming and migratory abilities in vitro [126]. Aside from directly impacting YAP and its downstream targets, verteporfin treatment also reduces ROCK2 protein levels, and FAK protein and phosphorylation levels [126,135]. The exact mechanism behind the latter effects is unclear but all findings together suggest the interesting possibility that verteporfin could act to inhibit both YAP and the upstream signalling causing its activation. Very preliminary research employing an orthotopic xenotransplant model involving human Ewing sarcoma cells and severe combined immunodeficiency (SCID) mice suggests that verteporfin treatment combined with amputation decreases lung metastases with no toxicity effects. This indicates it could be a viable therapeutic option in other bone malignancies, such as OSA [136].

#### 6.2.2. Agave 

An in vitro study completed by Ferraiuolo and colleagues (2018) found that agave extract decreased YAP and TAZ protein levels through enhancing protein degradation in human OSA cell lines. Agave also decreased TAZ and YAP mRNA levels and their known downstream gene targets, possibly through modulating the levels of NF-kappa B p65 and NF-kappa B p50 [137]. NF-kappa B p65 form homodimers or heterodimers with NF-kappa B p50 to activate transcription, while NF- kappa B p50 homodimers repress transcription [138]. In general, agave treatment increased nuclear NF-kappa B p50, while decreasing nuclear NF-kappa B p65. At putative NF-kappa B sites in YAP and TAZ promoters, the levels of both NF-kappa B p50 and p65 are reduced. Mechanistically, this data showed that by inhibiting NF-kappa B recruitment to YAP and TAZ promoter sites, agave prevents YAP and TAZ transcription, and subsequent protein production, resulting in decreased expression of YAP/TAZ target genes and suppression of pro-tumourigenic phenotypes [137]. Additional research is necessary to identify what component within agave extract is responsible for Hippo signalling inhibition, and for systematic testing of this putative compound in OSA cell lines both in vitro and in vivo to demonstrate target specificity and anti-tumour effects.

#### 6.2.3. Repurposed Inhibitors: Dasatinib, Pazopanib, and Simvastatin

As mentioned previously, many pathways contribute to the activation of TAZ/YAP, either in cooperation or independently of Hippo signalling. Some of these signalling pathways already have known or developed inhibitors that could be also used to directly, or indirectly, impact TAZ/YAP. Oku and colleagues (2015) screened small molecules on breast cancer cells and found that dasatinib, statins and pazopanib inhibited TEAD activity, and increased TAZ/YAP phosphorylation [139].

Dasatinib is an FDA-approved small molecular inhibitor with demonstrated activity for several tyrosine kinases, such as Src family signalling, c-MET signalling and the ability to modulate the localisation of TAZ/YAP, and actin reorganisation [139,140]. Activated Src signalling has been shown to increase TAZ/YAP transcriptional activity through reducing LATS1/2 activity and subsequent TAZ/YAP phosphorylation [141]. In vitro, dasatinib has been demonstrated to inhibit migration, invasion and viability in both human and canine OSA cells lines [142,143]. A small canine patient trial that tested dasatinib alone or in combination with adjuvant chemotherapy demonstrated treatment tolerability in both cases. When dasatinib was combined with a standard of care regime involving limb amputation followed by carboplatin, the survival time was prolonged when compared to historical controls [143,144]. A single human OSA patient case study was recently completed, but the treatment was combined with ceritinib, another pan-kinase inhibitor, making it difficult to discern the efficacy of dasatinib alone [145]. The treatment combination was well tolerated with only small side effects; however, the patient did eventually succumb to liver metastasis.

Pazopanib is a tyrosine kinase inhibitor of VEGF, platelet derived growth factor (PDGF) and stem cell factor (SCF)/c-KIT signalling, which is approved for the treatment of advanced soft tissue sarcoma. Pazopanib decreases the nuclear localisation of TAZ/YAP through enhancing its proteasome-mediated degradation [139]. So far, the clinical studies on the use of pazopanib to treat OSA report of conventional therapy-refractory patients with limited number of cases. However, the results are promising. One study, reporting the use of pazopanib in 15 metastatic OSA patients, showed that it was relatively well tolerated and it had a clinical benefit in 60% of the patients (partial response or stable disease), but the duration of the response was short (median PFS = 6 months) [146]. An independent study with a refractory bone sarcoma population of 19 patients showed comparable results; 68% patients showed clinical benefit [147]. However, little is known about the clinical benefit of pazopanib in comparison to conventional therapies.

Statins are a family of inhibitors of 3-hydroxy-methylglutaryl CoA reductase that are still under investigation for the treatment of various cancers and have shown to impair TAZ/YAP nuclear translocation [148]. Simvastatin in particular has been shown to inhibit cell growth and cell cycle progression, while increasing apoptosis in human OSA cell lines [149]. The pro-apoptotic capabilities of simvastatin are possible through increasing the activity of AMP-activated protein kinase (AMPK) and MAPK signalling, which can be enhanced with metformin treatment. Simvastatin alone inhibited in vivo growth of OSA tumours when mice were fed a fat-free diet, and the addition of metformin significantly enhanced its anti-tumour effects [150].

Although the aforementioned studies describing repurposed inhibitors provide interesting results, it is unclear if the effects are due to an inhibition of TAZ/YAP as it was not directly measured in the in vitro studies or in the clinical cases mentioned above. The original screening completed by Oku and colleagues (2015) was completed in breast cancer cells and needs to be completed in OSA to determine if the effects observed are TAZ/YAP-mediated, or simply due to the impact of the compounds on other signalling pathways. It will be also important to assess the signalling profiles of tumours in future OSA clinical trials, to address whether patient response correlates with TAZ/YAP activity. Drug repurposing is a beneficial avenue to explore as the safety profile of these compounds in humans are already known.

## 7. Myocardin-Related Transcription Factor-A/-B (MRTF-A/-B) (Rho/MRTF/SRF Signalling)

Similar to TAZ/YAP, the subcellular localisation and activity of MRTFs have been demonstrated to be mediated by cytoskeletal dynamics. MRTFs are normally bound to G-actin. Upon focal adhesion assembly and ROCK-mediated actin polymerisation, G-actin is incorporated to make F-actin causing the dissociation of MRTFs from G-actin, allowing their translocation to the nucleus where they can bind to serum-response factors (SRF) [151,152]. This interaction causes a subsequent increase in SRF-related genes which include cytoskeletal and focal adhesion components [153]. siRNA-mediated depletion of MRTF-A leads to a significant decrease in the levels of focal adhesion-associated proteins, namely, paxillin, vinculin, zyxin and in the activation of FAK [154].

### 7.1. MRTFs in OSA Mechanotransduction

To the best of our knowledge, little is known about MRTF-A/-B in OSA as our literature search only found one study conducted in human OSA. Dai and colleagues (2019) seeded MG63 human OSA cells on soft, medium, and rigid polyacrylamide hydrogels to determine the impact of rigidity on MRTF-A and epithelial-mesenchymal transition (EMT) protein levels and localisation. MRTF-A (total and nuclear), vimentin, snail, fibronectin, and MMP9 protein levels were increased with increasing hydrogel stiffness. MG63 cells also had greater migration speed when seeded on a rigid hydrogel stiffness [155]. These results suggest that in response to a stiff environment, MRTF-A is activated and promotes EMT in OSA, in agreement with findings in normal and malignant epithelial cells [156]. As the activation of MRTF can lead to cytoskeletal and extracellular matrix changes through the increase of fibronectin, targeting the Rho/MRTF/SRF signalling may be an attractive target as it would inhibit both ‘outside-in’ and ‘inside-out’ signalling. Fibronectin has also been shown to increase nuclear YAP accumulation, leading to the possibility that MRTF-induced fibronectin expression could also mediate YAP activity [157]. In addition, a crosstalk between MRTF-SRF and YAP-TEAD signalling in cancer-associated fibroblasts and breast cancer cells have been shown to promote metastasis [158,159]. This suggests targeting both MRTF and YAP could be beneficial in inhibiting mechanosignalling in cancer.

### 7.2. Inhibitors of Rho/MRTF/SRF Signaling

There are several small molecule inhibitors available that target the Rho/MRTF/SRF pathway, including CCG-1423, CCG-203971, and CCG-232601. Despite CCG-1423 having anti-invasive effects in prostate cancer cells, the compound was cytotoxic and had undesirable side effects in mice. This led to the creation of a 2nd generation inhibitor, CCG-203971 [160,161], which was found to be well tolerated in mouse models. The most recent inhibitor, CCG-232601 has a greater solubility and achieves higher concentration in plasma, suggesting it may be metabolically stable [162]. Only CCG-203971 has been tested in OSA cell lines, where it was shown to prevent the nuclear localisation of MRTF. Treatment of MG63 cells with CCG-203971 decreased the nuclear and total protein levels of MRTF-A, vimentin, snail, fibronectin and MMP9 in a stiff environment [163]. Recent studies showed that treatment with CCG-203971 decreased cell migration, even in a stiff environment [155]. Further tests using in vivo models of metastatic progression will be informative as to the potential translation of this drug to the clinic. 

## 8. Ezrin

Ezrin is a member of the ERM (ezrin, radixin, moesin) family of proteins and has a unique protein structure enabling it to bind to both, the actin cytoskeleton and the plasma membrane [164,165]. Through the FERM domain (four point one, ezrin, radixin, moesin) in its N-terminus, ezrin has been shown to bind to CD44, CD43, intercellular adhesion molecule-2 (ICAM-2) and integrins, while its C-terminus can bind to F-actin [166,167]. Ezrin can exist in two different confirmations, open (active) and closed (inactive). In the closed state, the N-terminus and C-terminus bind to each other through intramolecular interactions, preventing its binding to F-actin [168]. To become active, phosphatidylinositol 4,5-bisphosphate (PIP_2_) or S100P proteins binds to the FERM domain, allowing for its dissociation. This allows for the subsequent phosphorylation at threonine residue 567 (T567) which is mediated by protein kinase C (PKC) and ROCK; this phosphorylation is essential for ezrin’s activation and ability to bind to F-actin [169].

Ezrin is highly expressed in sarcomas and was shown to associate with a shorter disease-free interval in both canine and human OSA patients [170,171]. A highly metastatic variant of murine OSA cells is enriched for ezrin and in vivo mouse models demonstrated that ezrin promotes a survival effect upon cancer cell’s arrival to the lung, possibly through the activation of MAPK signalling [172]. Interestingly, the conformation of ezrin was also shown to be important for metastatic lung colonisation by OSA cells. While total ezrin levels remain uniform in the metastatic lesion upon seeding, the activation of ezrin appears to be dynamic and occurs only at the periphery of the metastatic lesion. Furthermore, both phosphodefective (T567A) and phosphomimetic (T567D) ezrin mutants are unable to form metastases in the lung, even 100 days post tail vein injection [173]. This suggests that both open and closed conformations of ezrin, and transitions between these states are important at different times within the metastatic cascade.

### 8.1. Ezrin and Mechanotransduction

Although there are no studies to date that have directly investigated the role of ezrin in mechanotransduction in OSA cells, there are several lines of evidence to suggest that this relationship exists in other normal and cancerous cell types. Fluid shear stress (FFS) stimulation of human placenta cells promoted the formation of microvilli and an increase of internal Ca^2+^ through calcium ion channel TRPV6. The increase in Ca^2+^ enhanced phosphorylation of Akt and ezrin, providing evidence that mechanosensitive microvilli could lead to an activation of ezrin [174]. Aside from indirect mechanical signalling impacting ezrin activation, ezrin itself could be a mechanotransducer. Ezrin’s ability to bind to the cell membrane and F-actin means it is in a good position to be able to receive and respond to extracellular stimuli. Some evidence suggests this is case; studies that have depleted ezrin levels or decreased phosphorylation levels demonstrated a decreased stress fibre density and limited focal adhesions or membrane tension and cytoskeletal organisation, respectively [175,176]. Along these lines, fibroblasts transfected with various ezrin mutants found that the phosphomimetic mutant had more spread tubulin fibres, and increased vimentin and actin fibre length. Lastly, although the cortical stiffness was decreased in phosphomimetic cells, the cytoskeleton stiffness was increased when compared to wild-type transfected cells. These two findings appear conflicting but are both in line with increased migratory abilities in cell transfected with this mutant [177].

Because of ezrin’s ability to modulate actin dynamics and promote metastasis in in vivo models of various cancers, small molecule inhibitors have been developed to target ezrin. Aside from ezrin itself being important for migration and metastasis, ezrin can modulate the levels and localisation of YAP in skin fibroblasts, as well as pancreatic and hepatocellular carcinoma cancer cells. The positive effect of ezrin on YAP can be direct, as seen in skin fibroblasts [178], or through the activation of other signalling pathways such as Akt/mTOR, as seen in pancreatic cancer cells [179]. Alternatively, the T567-phosphorylated form of ezrin can suppress Hippo signalling, as seen in hepatocellular carcinoma [180]. Although none of these mechanisms have been specifically demonstrated in OSA; these evidences, along with ezrin’s ability to modulate actin dynamics, and promote OSA metastasis in vivo, provide sufficient rationale to target ezrin signalling in OSA.

### 8.2. Inhibitors of Ezrin

As the phosphorylated (active) form of ezrin has been associated with pro-migratory and pro-invasive behaviour in both canine and human OSA, small molecule inhibitors have been focused on disrupting the phosphorylation site of ezrin and its ability to bind to F-actin. A small molecule library screen to determine compounds that would directly bind to ezrin identified two molecules, NSC305787 and NSC668394. In vitro assays found that these compounds work by directly binding to ezrin to inhibit phosphorylation; although NSC305787 can inhibit PKC kinase activity at higher doses, this is not the case for NSC668394 [181]. The invasion capacity of murine OSA cells expressing high levels of ezrin was blunted by treatment with either compound in vitro, which also significantly decreased metastatic growth in ex vivo lung cultures, with NSC305787 showing slightly greater anti-metastatic effects compared to control. Lastly, in vivo tail vein injection of OSA cells followed by treatment with either inhibitor demonstrated that NSC305787 prolongs survival compared to the vehicle control. Further studies performed using transgenic models of OSA (Osx-Cre^+^ *p53*^fl/fl^ *pRB*^fl/fl^) exhibited similar results, as the incidence of lung metastasis was significantly reduced with NSC305787 treatment, a compound that also reached the more favourable plasma concentrations as compared to NSC668394 [182].

Because of the similarity in structure of NSC305787 to quinoline-based antimalarial treatments, several anti-malarial agents have been tested and were demonstrated to have anti-ezrin activity in a Medicines for Malaria Venture (MMV) screen. Several candidate compounds that were found in this screen (MMV667492, MMV020549, MMV666069, MMV665877) decreased cancer cell motility in vitro and substantially decreased lung metastatic growth in an ex vivo lung culture compared to NSC305787. Interestingly, all the compounds tested increased the expression of phosphorylated (T567) ezrin, the most modest being MMV667492, which caused no changes in total phospho-ezrin levels [183]. This suggest at least two possibilities, (i) that by enhancing phosphorylation these compounds might interfere with the putative dynamic switch between open and close conformation required for optimal metastatic growth, suggested by the previously discussed study with phosphomimetic and phosphodefective mutants [173]; or (ii) that these anti-malarial compounds could exert anti-ezrin activities through other means, perhaps preventing phosphorylation-independent protein–protein interactions or impacting other phosphorylation sites (i.e.,: Tyr354) that have been described for ezrin [184,185]. Further studies need to be conducted to elucidate how targeting these functions could impair the pro-metastatic capabilities of ezrin.

## 9. Nuclear Mechanotransduction: Factors for Force Transmission and DNA Repair

Aside from the most studied mechanotransducers, such as those mentioned in Section 6, Section 7 and Section 8 (e.g., integrins, focal adhesion components and downstream signalling molecules), less documented but equally relevant nucleoskeleton components must be also considered as promising therapeutic targets. The nucleus is a highly dynamic unit that can transmit force through various molecules and molecular complexes, such as lamins, nuclear actin, and the linker of the nucleoskeleton and cytoskeleton (LINC) complex [186]. Apart from mediating mechanosignalling, these nuclear molecules/complexes participate in cellular processes that notably contribute to cancer progression, namely DNA repair, transcription, and replication. Nuclear mechanostransducers participate in these processes by serving as scaffolds for the enzymes and other proteins that mediate them [187]. In terms of OSA, lamin B1 was shown to regulate nucleotide excision repair (NER) in response to ultraviolent damage, by altering the expression level of genes associated with NER [188]. Recent reports suggest that OSA cells utilise DNA repair mechanisms, such as NER, to circumvent DNA-damaging chemotherapeutics, such as cisplatin [189]. One attractive treatment strategy could include targeting NER factors. Fanelli and colleagues (2020) recently found that two compounds, NSC130813 (NERI02; F06) and triptolide, which inhibit DNA repair through binding of DNA repair proteins, enhance sensitivity to cisplatin in U2OS and Saos2 cells [190]. Understanding how these factors function in the context of mechanotransduction could aid in the success of NSC130813 (NERI02; F06) and triptolide in treating refractory OSA patients.

## 10. Unanswered Questions and Possible Avenues for Future Research

Compared to epithelial malignancies, research on mechanical influences in osteosarcomagenesis and progression is still in its infancy. All the evidence discussed in this review is solely based on in vitro experiments utilising a limited number of cell lines and variable ex-vivo or animal models, making the results extremely difficult to extrapolate. There are a considerable number of unknowns that will need to be addressed in order to fully understand the mechanical landscape of OSA, some of which are briefly discussed below:

1. How does the mechanical bone and lung microenvironment change during osteosarcoma progression? Panciera and colleagues (2020) recently found that constitutive oncogenic signalling, specifically by Kirsten rat sarcoma viral oncogene homolog (K-RAS) or HER2, alone, is not enough to reprogram mammary cells into tumour-initiating cells but might additionally require a stiffer-than-normal mechanical microenvironment [191]. Given that calcified collagenous bone is already a stiff microenvironment, it is unclear how, and if, the osteoid production by tumour cells contributes to the stiffness of the microenvironment and what are the implications of such contribution to tumour development and progression. This is also unknown for the lung microenvironment; however, there is indirect evidence to suggest that stromal-tumour interactions within the lung, possibly resulting in enhanced ECM stiffness and subsequent mechanotransduction, favour lung colonisation. Fibroblast growth factor receptor signalling can increase the production of fibronectin by stage-specific embryonic antigen-4 positive (SSEA4^+^) OSA stem cells and causes a fibrogenic reprogramming. This reprogramming creates a fibrotic-like environment and provides a survival advantage for lung metastasis growth, but not primary bone lesion growth [192]. Although not directly investigated in this study, Liu and colleagues (2015) reported that normal lungs have a median shear modulus of 0.59 kPa, while the fibrotic regions of idiopathic pulmonary fibrosis lungs have a median shear modulus of 5.16 kPa [193]. It will be interesting for future research to determine if this fibrogenic reprogramming of OSA stem cells impacts the mechanical properties of the microenvironment to activate tumour-promoting mechanosignals.
2. Do mechanotransduction mechanisms vary in early stage as compared to advanced stage tumour cells? A majority of the research mentioned in this review only included studies that utilised primary OSA cell lines, which makes it difficult to understand any differences in how primary and metastatic OSA cells respond to mechanical cues. Future research could compare paired primary- and metastatic-derived OSA cells in microenvironments with varying stiffnesses to assess the mechanical signalling pathways that are activated and the differences, if any, in functional cell responses.
3. Can we identify tumour mechanotransduction signatures to tailor therapy? Most studies that have explored mechanical signatures in epithelial cancer tissue include profiling the stiffness of cancer biopsy tissue ex vivo using indentation techniques [194,195], or using shear wave elastography (SWE) to evaluate the stiffness of the cancer tissue itself and/or surrounding lymph nodes to determine malignancy [196,197]. These methods are helpful as diagnostic and prognostic tools, but do not provide insight into what is actually driving the mechanical signalling. Future studies should explore the possibility of developing a ‘mechanical signature’ in OSA to better predict mechanical signalling and cancer progression. One possible avenue is determining the presence of mechanosignalling mediators in plasma or serum samples from OSA patients. Given the importance of ECM remodelling in cancer, Andriani and colleagues (2018) explored the biomarker potential of collagen type X alpha 1 (COL10A1), collagen type XI alpha 1 (COL11A1) and collagen-binding molecule, secreted protein acidic and rich in cysteine (SPARC) in plasma of lung cancer patients [198]. The levels of COL10A1 and SPARC were significantly higher in lung cancer patients compared to healthy controls. Aside from looking at ECM proteins in plasma, future research could also explore the presence and abundance of mechanical signalling machinery in extracellular vesicles, which are present in high quantities in the circulation of cancer patients and are known to be able to interact with recipient cells and modify behaviour [199,200].
4. How do genomic aberrations in OSA impact mechanosignalling? The karyotype of OSA is notoriously complex; genomic analysis of OSA tumours from both human and canine samples show multiple copy number variations and structural variants of certain genes [201,202]. This genomic heterogeneity between and within OSA tumours will undoubtedly challenge the way we understand mechanosignalling and our ability to target it in a patient context. It is imperative that we use a strategic approach to understand how certain mutations can attenuate or potentiate mechanosignalling. One possible way to approach this is to generate and analyse robust datasets from sequenced human and canine OSA tumours and find commonly altered genes [201]. We can then map signalling networks and possibly identify one or more mechanotransduction mediators within these networks. Experiments could use cell lines that bear these mutations or use genetically modified models to create similar mutations and explore their response to mechanical stimuli in both 2D and 3D culture models. Such a link between a mutated gene and mechanotransduction is exemplified by insulin-like growth factor-1 receptor (IGF1R), which has been shown to bear somatic mutations in human OSA [203]. Tahimic and colleagues (2016) found that with mechanical stimulus, IGF1R undergoes activation in an integrin-dependent fashion to activate downstream signalling molecules, such as FAK. In turn, FAK can also mediate response to ligand-dependent IGF1R activation [204]. Given this relationship between IGF1R and mechanical stimulus, further research will be needed to understand how, and if, somatic mutations in IGF1R could potentiate the FAK signalling cascade. Another avenue to explore is understanding how and if mechanosignalling contributes to genomic instability. The mechanical environment can impact cellular processes such as mitosis, chromosome segregation and chromosome architecture, all of which can contribute to abnormal genotypes [205,206]. This really raises the which came first, the chicken-or-the-egg question: do genomic aberrations potentiate mechanosignalling or are genomic aberrations the result of mechanical forces in the environment? In order to address these questions, it is imperative that we utilise a comprehensive human, canine and murine OSA model approach to permit the assessment of clinical relevance side-by-side proof-of-concept experiments, resulting in faster advances in OSA treatment [207].

## 11. Conclusions

While the role of cellular signalling pathways in OSA progression have been well investigated, the research on the role of mechanical signalling in OSA has been lacking. Preliminary research conducted to date has demonstrated that the mechanical properties of OSA cells changes with increasing malignancy and that OSA cells utilise several mechanisms (TAZ/YAP, MRTF and Ezrin) to respond to mechanical cues. Robust studies using clinically relevant mouse models and comparative oncology approach using canines as a model are imperative to enhance our understanding of mechanobiology and create suitable molecular targeted therapy for advanced OSA.

## Figures and Tables

**Table 1:** Summary of literature included in this review that characterised the mechanical properties of normal bone and osteosarcoma (OSA) cells.

Scope of Paper	Cell Line(s)	Model	Key Findings
Compared mechanical properties of individual mesenchymal stem cell (MSc), osteoblast (NHost) and OSA cells (MG63) [81]	MSc NHost MG63	2D	MG63 are smaller, thicker, less stiff and had a rougher membrane compared to MSc and NHost
Characterised the mechanical properties of U2OS during interphase and telophase of mitosis in two different regions within the cell [90]	U2OS	2D	U2OS stiffer overall in interphase; periphery of the cell stiffer than nuclear region during interphase and telophase
Compared the mechanical properties between two paired primary and metastatic OSA cells [82]	SaO2/LM5 HuO9/M132	2D	Low metastatic cells had a greater spreading area, focal adhesion count and density; other measured parameters were inconsistent between pairs
Exposed U2OS cells to different degrees of confinements to determine changes in mechanical properties [86]	U2OS	1D microlines + Y-shaped PDMS device	U2OS cells soften and YAP is cytoplasmic during confinement in PDMS model but not 1D microline model
Characterised cell morphology, size and traction forces of bone cells at different differentiation stages [87]	MSC dMSC osteoblasts osteocyte	2D	Osteoblasts and osteocytes had larger surface area; cell circularity, inverse aspect ratio and traction force generation positively correlated with differentiation

PDMS—polydimethylsiloxane; MSC—mesenchymal stem cell; dMSC—differentiated mesenchymal stem cell.

**Table 2:** Summary of the literature included in this review on how Ewing sarcoma and OSA cells respond to mechanical stress.

Scope of Paper	Cell Line(s)	Model	Key Findings
Isolated tumour cells from human OSA patient and cultured on different substrate rigidities [91]	Primary human OSA cells	2D collagen-coated PA gels	Cells cultured on 55 kPa was most compatible for growth, cell survival and generated most traction forces
Cultured sarcospheres in PEGDA gels with various rigidities to determine most optimal environment [92]	U2OS	PEGDA gels	50 kPa was the most optimal PEGDA gel to form CD133^+^ and CD44^+^ sarcospheres
Investigated the role of integrin beta 1 and FAK signalling in response to mechanical stimulation [93]	MG63	2D + mechanical stimulation	Increase in integrin beta 1, pFAK and pERK protein levels with mechanical strain; blockade of integrin beta 1 blunted increase in pFAK and pERK with mechanical stimulation
Determined how normal osteoblast and osteosarcoma cells respond to microenvironments with varying adhesion ligand density and stiffness [94]	Normal osteoblasts MG63	PEGDA/GelMa hydrogels	Normal bone cells more responsive to adhesion ligand density of the ECM, while OSA cells more responsive to ECM stiffness; increasing stiffness led to an increase in FA signalling proteins, pro-tumorigenic mRNAs and in vivo tumorigenicity for OSA cells
Explored the effects of mechanical strain on TN-C FNIII A1 mRNA and protein levels [95]	MG63	3D collagen + 0.2 Hz cyclic strain	Increase in TN-C FNIII A1 mRNA and protein upon mechanical strain; silencing of downstream mTOR signalling (4E-BP1 and S6K1) blunts these effects

PA—polyacrylamide hydrogel (varying ratios of acrylamide and bis-acrylamide allows changes in substrate rigidity); PEGDA—polyethylene glycol diacrylate (manipulation in hydrogel crosslinking and density allows changes in stiffness); GelMA—methacrylated gelatin (manipulation in composition allow changes in ligand density).

## Abbreviations

OSA	osteosarcoma
PGE_2_	prostaglandin E_2_
COX2	cyclooxygenase 2
ATP	adenosine triphosphate
cAMP	cyclic adenosine monophosphate
PKA	protein kinase A
PI3K	phosphatidylinositol 3-kinase
GSK3	glycogen synthase kinase 3
CX43	connexin 43
TRPV4	transient receptor potential vanilloid subfamily member 4
ER	endoplasmic reticulum
P2R	P2 purinergic receptor
PLC	phospholipase C
MLCK	myosin light chain kinase
EV	extracellular vesicle
RANKL	receptor activator of nuclear factor kappa-B ligand
OGN	osteoprotegerin
FAK	focal adhesion kinase
Rho	Ras homologue
GTPase	guanosine triphosphatase
LEF/TCF	lymphoid enhancer-binding factor/T-cell factor
p38 MAPK	p38 mitogen activated protein kinase
NF-kappa B	nuclear factor kappa B
BMP	bone morphogenetic protein
SMAD	small mothers against decapentaplegic
ALP	alkaline phosphatase
OCN	osteocalcin
COL1	collagen 1
JNK	Jun N-terminal kinase
HER2	human epidermal growth factor receptor 2
IGF1	insulin-like growth factor 1
mTOR	mammalian target of rapamycin
ECM	extracellular matrix
LOX	lysyl oxidase
ROCK	Rho-associated coiled-coil containing kinase
kPa	kilo pascals
PEGDA	polyethylene glycol diacrylate
ERK	extracellular signal-regulated kinase
HIF1-alpha	hypoxia inducible factor 1 alpha
VEGF	vascular endothelial growth factor
MMP	matrix metalloproteinase
FNIII A1	fibronectin III A1
TN-C	tenascin C
4E-BP1	eukaryotic translation initiation factor 4E (eIF4E)-binding protein 1
S6K1	p70 ribosomal protein S6 kinase 1
P4H	prolyl 4-hydroxylases
PLOD	procollagen-lysine, 2-oxoglutarate 5-dioxygenase
UPS	undifferentiated pleomorphic sarcoma
TAZ	transcriptional co-activator with a PDZ-binding motif
YAP	yes-associated protein
MRTF-A/-B	myocardin-related transcription factor-A/-B
MST1/2	mammalian Ste20-like kinase
LATS1/2	large animal tumour suppressor 1/2
TEAD	TEA domain family member
CYR61	cysteine-rich angiogenic inducer 61
CTGF	connective tissue growth factor
CCND1	cyclin D1
PCL	poly(ε-caprolactone)
SCID	severe combined immunodeficiency
PDGF	platelet derived growth factor
SCF	stem cell factor
AMPK	AMP-activated protein kinase
SRF	serum-response factors
EMT	epithelial-mesenchymal transition
ERM	ezrin, radixin, moesin
FERM	four point one, ezrin radixin, moesin
ICAM-2	intercellular adhesion molecule 2
PIP_2_	phosphatidylinositol 4,5-bisphosphate
PKC	protein kinase C
LINC	linker of the nucleoskeleton and cytoskeleton
NER	nucleotide excision repair
K-RAS	Kirsten rat sarcoma viral oncogene homolog
SSEA4+	stage-specific embryonic antigen-4 positive
SWE	shear wave elastography
COL10A1	collagen type X alpha 1
COL11A1	collagen type XI alpha 1
SPARC	secreted protein acidic and rich in cysteine
IGF1R	insulin-like growth factor-1 receptor
